# Microscopic Investigation of Reversible Nanoscale Surface Size Dependent Protein Conjugation

**DOI:** 10.3390/ijms10052348

**Published:** 2009-05-20

**Authors:** Kazushige Yokoyama, Hyunah Cho, Sean P. Cullen, Matthew Kowalik, Nicole M. Briglio, Harold J. Hoops, Zhouying Zhao, Michael A. Carpenter

**Affiliations:** 1 Department of Chemistry, The State University of New York at Geneseo, 1 College Circle, Geneseo, NY 14454, USA; E-Mails: hc7@geneseo.edu (H.C.); spc8@geneseo.edu (S.P.C.); mk16@geneseo.edu (M.K.); nmb11@geneseo.edu (N.M.B.); 2 Department of Biology, The State University of New York at Geneseo, 1 College Circle, Geneseo, NY 14454, USA; E-Mail: hoops@geneseo.edu (H.J.H.); 3 College of Nanoscale Science and Engineering, University of Albany-SUNY, 255 Fuller Road, Albany, NY 12203, USA; E-Mails: zzhao@uamail.albany.edu (Z.-Y.Z.); mcarpenter@uamail.albany.edu (M.A.C.)

**Keywords:** amyloid beta, fibrillogenesis, Alzheimer’s disease, ovalbumin, gold nanoparticles, AFM, TEM

## Abstract

Aβ_1–40_ coated 20 nm gold colloidal nanoparticles exhibit a reversible color change as pH is externally altered between pH 4 and 10. This reversible process may contain important information on the initial reversible step reported for the fibrillogenesis of Aβ (a hallmark of Alzheimer’s disease). We examined this reversible color change by microscopic investigations. AFM images on graphite surfaces revealed the morphology of Aβ aggregates with gold colloids. TEM images clearly demonstrate the correspondence between spectroscopic features and conformational changes of the gold colloid.

## 1. Introduction

Nanoscale materials have revolutionalized the function and use of materials in many fields including the biomedical field. One of the fascinating features of nanobiomaterials is that they are able to characterize functions of biomolecules placed *in vivo* or *in vitro*. Our group’s focus is the biological function of the nanoscale surface of metal colloids. These metal colloids can play key roles in monitoring biological functions under interfacial conditions and ultimately provide us the characterization of the biological molecules on human membranes or cells. The conjugation of colloidal gold with biospecific macromolecules has been extensively studied [1–13], and particularly a series of studies showed spectroscopic techniques were able to monitor conformational change on the gold colloidal nanoparticles [14]. The proteins immobilized at an interface are expected to display different properties than their counterparts in bulk solutions [15–18]. Understanding the interactions of the proteins on the surface is crucial to designing a bio-composite device, while directly monitoring the specific property attributed to the interfacial environment is a very challenging issue.

**Figure 1.**
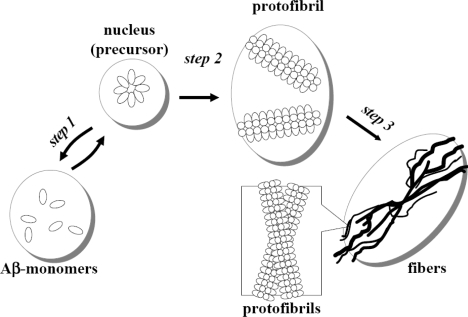
A model of fibrillogenesis. In step 1, monomeric Aβ forms nuclei from which protofibrils emanate (step 2). These protofibrils give rise to fill-length fibers (step 3).

We have been investigating the conjugation of amyloid β protein (Aβ) over gold nanocolloid especially focusing on its connection to a mechanism of fibrillogenesis, which is associated with Alzheimer’s disease [19,20]. The cellular interfaces play a major role in the aggregation of amyloid peptides *in vivo* [21] and the approach of using the gold nanoparticles surfaces to mimic cellular surfaces is very effective. Pathologically, a key hallmark of the neuritic and cerebrovascular amyloid in Alzheimer’s disease is the formation of insoluble fibrillar deposits of Aβ as both diffuse and senile amyloid plaque that invades the brain’s seat of memory and cognition before it spreads to other regions [22–25]. In Figure 1, the polymerization process and progresses of monomeric Aβ to form fiber-like aggregates are illustrated. This entire process is known as fibrillogenesis. A key step of the fibrillogenesis is the nucleation, in which a certain number of monomer units construct a seeding aggregate [26,27] leading into the amyloid fibril lattice [28]. The fibril lattice eventually elongates to form the fibril. The initial stage of an entire fibrillogenesis has been regarded as a crucial and important step because it is a key onset for the aggregation to follow. However, the structure of the precursor is not fully understood.

Fibrillogenesis is thought to take place at the interface of human membrane or blood cells, and studying the structure of the intermediate at an interface is crucial to understanding the process. Until now, limited studies have been performed to investigate this phenomenon due to the theoretical [45–47] and experimental [48–54] complexity of secondary and tertiary protein structures forming from non-native conformations at an interface. This is a key step of fibrillogenesis, yet most *in vitro* studies overlook this step and focus on the aggregation of Aβ prepared in solution. At the air-water interface, Aβ self assembles into an organized nanostructure with no regular secondary structure [29]. This implies that large conformational changes, and thus high activation energies, are required for monomeric Aβ to bind to nascent fibrils [30]. It may be plausible then, for aggregation with lower activation energy to result from Aβ adsorbed on the *in vivo* surface. While peptides form α-helical structures in solutions of negatively charged micelles and teflon particles [31–34], the Aβ protein forms β-sheet structures on hydrophobic graphite surfaces [35] and at air–water interfaces [36]. When fluorinated nanoparticles (with a diameter of about 2 nm) were used, the β-sheet structure was heavily influenced. Thus, self assembly over the nanoscale interfacial environment plays a key role in fibrillogenesis [37]. This conformation of the Aβ self assembly on the membrane surface may be seen when Aβ conjugates on the surface of gold colloidal nanoparticle. The three dimensional network of the conjugated proteins can be investigated by understanding the protein interactions on different surface sizes. Studying Aβ on gold colloid is particularly interesting since amyloid fibrils attached to gold nanoparticles can be destroyed, without harming healthy cells, when exposed to weak microwave fields [38]. An early onset of Alzheimer’s disease is believed to be peptide conformational changes and aggregation [39]. A drug designed to control and stabilize the Aβ secondary structure would be a plausible therapeutic approach to prevent these initial steps.

In this study, we investigated Aβ at an interfacial environment on the surface of gold colloidal nanoparticles to mimic a structure similar to that of Aβ located at the interfacial environment on the surface of brain or membrane cells. It should be noted that the metal colloidal surface environment is not the same as that of physiological conditions. However, it allows us to explore nanoscale dimensionality of protein structure at an interface by studying Aβ’s structure on a size-controlled interfacial environment. We recently discovered that pH-induced reversible self-assembly of Aβ on spherical gold nanoparticles is sequence and gold colloid size dependent [19]. Among several tested Aβ sequences, a reversible process took place only with Aβ_1–40_ with 20 nm gold colloidal nanoparticles. However, ovalbumin coated gold colloid exhibited reversibility for all tested sizes of gold colloids ranging from 5 nm to 100 nm sizes [20]. This reversibility was seen spectroscopically and visually between pH 4 and pH 10, exhibiting a color change from blue to pink, respectively. Quite significantly, this reversible process implies a correspondence to the initial reversible stage of fibrillogenesis (step 1 in Figure 1) and may involve the structural conformation of its intermediate. The color change of the solution is attributed to a structural modification of the protein on the surface of colloids.

While the spectroscopic study implies the possibility of conformational changes of protein conjugated on the gold colloids, no direct evidence of conformational change was identified. Specifically, the reversibility needs to be fully examined as the repetition number increases. In general, the gold colloid is considered to form aggregates at the lower pH and is dispersed at the higher pH. Our spectroscopic approach, however, lacks a means of directly observing the conformation. A microscopic approach enables us to confirm what the conformation at a given spectroscopic feature. It provides conformational information of the conjugated protein, thus giving us further insight into the intermediate structure of Aβ at the interfacial environment.

## 2. Results and Discussion

### 2.1. The Effect of Protein Conjugation to the Spectroscopic Feature of the Gold Colloid

**Figure 2.**
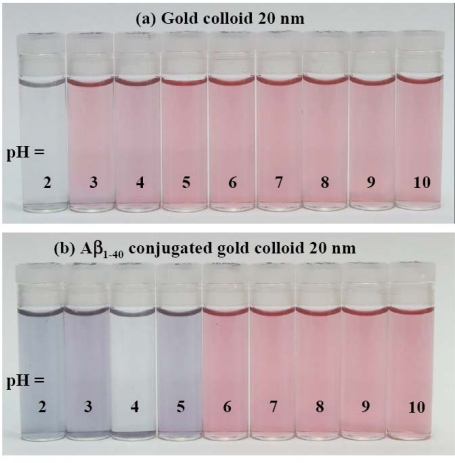
Visual evidence of the effect of pH on a colloidal gold nanoparticle solution. (a) The color of the solution of gold colloidal nanoparticle with size 20 nm. (b) The color of 20 nm colloidal gold nanoparticles coated with Aβ_1–40_.

Figure 2 provides visual colorimetric observation of the effect of pH on the bare gold nanoparticle or gold nanoparticles coated with Aβ_1–40_. The bare gold nanoparticle solutions in Figure 2a show almost no change from the original color except for pH 2, whereas the Aβ_1–40_ coated particles in Figure 2b display color variation around pH 5 or lower pHs.

**Figure 3.**
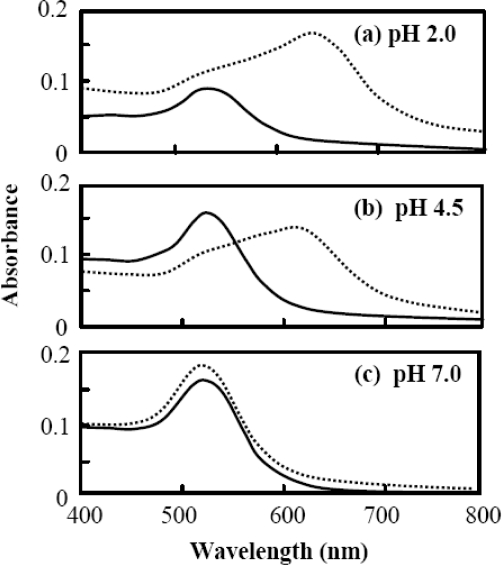
The absorption spectra of gold colloidal nanoparticles with a diameter of 20 nm (thick line) and Aβ_1–40_ mixed with gold colloid (dotted line) for (a) pH = 2.0, (b) pH = 4.5, and (c) pH = 7.0.

The representative absorption spectra are shown in Figure 3 for 20 nm gold colloids and Aβ_1–40_ coated 20 nm gold colloids. All of the absorption bands were fit with a Gaussian profile using the peak-fit-module of ORIGIN (Version 7.0) in the range of 400 to 800 nm. When the band component included multiple parts, the peak position, λ_peak_, was determined by:

(1) $$\lambda_{peak}=\sum_{i=1}^{n}a_{i}\lambda_{i}$$

where λ_i_ and *a*_i_ represent the peak position and fraction of the i^th^ component band. Most of the bands observed in our study were fully analyzed with two components or one component with a large background band. The fraction *a*_i_ was determined by the fraction of the area (A_i_) of the band to the area of the total sum of the entire bands, *e.g*., *a*_1_ = A_1_/(A_1_ + A_2_) for the case of two bands. As shown in Figure 3a, the baseline of the absorbance was raised for Aβ_1–40_ mixed gold colloid. This is presumably due to the effect of light scattering contributed from aggregates.

**Figure 4.**
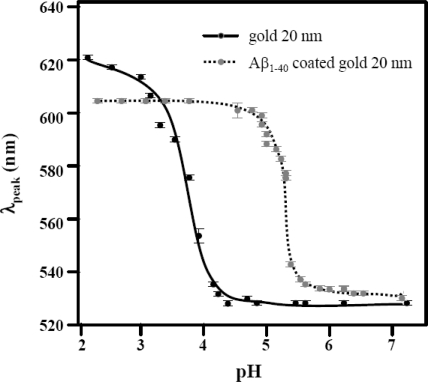
The peak position of the absorption spectrum in the region between 400 nm and 800 nm.

The peak shift of the absorption spectrum as a function of pH was monitored, and the position of the peaks as a function of the pH values were plotted as shown in Figure 4. For our analysis, an index showing color change as a function of pH was defined as pH_o_, which was extracted by an analytical formula characterized by a Boltzmann formula:

(2) $$\lambda_{peak}(pH)=[\lambda_{\text{min}}-\lambda_{\text{max}}]/\left\{1+\text{exp}\left[\left(pH-pH_{o}\right)/dpH\right]\right\}+\lambda_{\text{max}}$$

The λ_min_ and λ_max_ stand for the minimum and maximum of the band peak positions, respectively. The pH_o_ is the pH value at which λ_peak_ = (λ _min_ + λ _max_)/2. The *d*pH is defined as: *d*pH = (λ _max_ – λ_min_)/4λ _peak_^(1)^, where λ _peak_^(1)^ is the first derivative of the λ_peak_(pH).

The pH_o_ for 20 nm gold nanoparticles was determined to be 3.70 ± 0.07 and that for Aβ_1–40_ coated 20 nm gold colloid was 5.33 ± 0.01, which is close to the value of pI = 5.2. It is generally accepted that a protein conjugation onto the gold colloidal surface is observed *c.a.* 0.5 pH unit above pI value [40]. The values of *d*pH for 20 nm gold colloid and Aβ_1–40_ coated 20 nm gold colloid were 0.24 ± 0.07 and 0.08 ± 0.01, respectively. Since *d*pH is inversely proportional to λ _peak_^(1)^, the first derivative of the λ_peak_(pH), the smaller value in *d*pH indicates the higher λ_peak_^(1)^. The high λ_peak_^(1)^ of Aβ_1–40_ must imply that this sequence sensitively responds to the acidic condition and transforms conformation. Considering that a net charge of Aβ_1–40_ is none, higher responsiveness to pH change was surprising. We suspect the negatively charged segments reside in hydrophilic tail (i.e., 1^st^, 3^rd^, 7^th^, 11^th^ sequences - aspartic acid or glutamine) are responsible for interacting with acid leading to the unfolding of the conformation. The unfolded protein must be playing a key role to intermediate the gold colloids to form an aggregate. While the peak position at the higher pHs, λ _min_, were very similar for both 20 nm gold colloid and Aβ_1–40_ coated 20 nm gold colloid (526 ± 4 nm and 531 ± 1 nm), the peak position at the lower pH, λ _max_, was longer for 20 nm gold colloid (615 ± 6 nm) compared to that of Aβ_1–40_ coated 20 nm gold colloid (597 ± 1 nm).

### 2.2. The pH Induced Reversible Color Change of Protein Coated Gold Colloid

**Figure 5.**
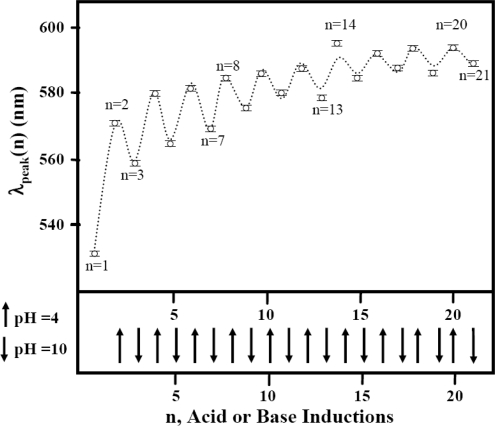
A demonstration of the color change reversibility seen in Aβ_1–40_ coated 20 nm gold colloidal particles (open circles). The upward and downward arrows indicate an injection of HCl and NaOH, adjusting the pH of the solution to pH 4 and pH 10, respectively. The dashed lines indicate the values predicted by Equation 3.

The reversibility of the color transition was examined by repeatedly varying pH values of the solution between pH 4 and 10 by addition of acid or base solutions to a sample mixture. The initial pH value was started at pH 7 for all solutions, and the pH value was changed from pH 7 to pH 4 by adding acid (HCl). The pH was shifted back to pH 10 by adding an appropriate amount of base (NaOH). A corresponding color change was clearly observed in only Aβ_1–40_ coated gold colloidal particles (Figure 5). However, the color change in the reversible process was not between pure blue and red, rather it was between purple and red.

We use the following analytical formula to characterize the property of the reversibility, the wave peak of each acid or base addition operation, λ_peak_(n):

(3) $$\lambda_{peak}(n)=A+B{\left(n-1\right)}^{C}+D\;\text{exp}\left(-(n-1)E\right)\text{cos}\left(n\pi \right)$$

where n indicates the operation of pH change. The n = 1 indicates the starting pH which is around pH 7. Odd numbers of n (n = 3, 5, 7,…) indicate an operation of acid addition, decreasing the pH of solution to pH 4, whereas even numbers of n (n = 2, 4, 6,…) show an operation of base addition to increase the pH of solution to pH 10. (The parameters A, B, C, D, and E were extracted and are shown in Table 1.) Equation 3 was devised to reproduce the spectral shift (the first term) and repetition change (the second term). In this equation an initial peak position at neutral pH (*i.e.,* λ_peak_(1)) is given by A – D, and the parameters B and C show the average wave peak position shift as pH varies between 4 and 10. In the second term, the parameter D and E imply amplitude and damping factor for the repetitive event, and the *sine* function was used to indicate peak up and down upon addition of acid and base. The values calculated by Equation 3 are effective only for each n value, not the values in between each n. Thus, the dotted line shown in Figure 5 is given only for the purpose of clarifying the repetitive trend.

The peak at pH 10 shifts gradually to 580 ± 1 nm from 528 nm as the repetition number of the pH change increased, while the absorption band at pH 4 appears around 585 nm, where it consists of two peaks where one centers around 528±1 nm and the other centers at 600 nm. The band around 528 ± 1 nm can be regarded as the free gold colloidal band and more free gold colloidal nanoparticle surfaces are produced as the pH changes are repeated. This can be because some Aβ can be desorbed from the gold surface as the pH decreased. Those freed Aβ may not have been re-adsorbed on the gold colloidal surfaces due to the aggregation between Aβ monomers. The investigation of the reversibility of the observed color change was an important part of our study, since the corresponding structural change can be the same structural change that exists in the reversible step of the fibrillogenesis where a cluster of monomer units form a nucleotide oligomer. The structural change enabling a reversible process must be attributed to a three dimensional hydrophilic network only possible in the Aβ_1–40_ sequence, since other segments did not indicate any structural reversibility as seen in gold colloidal nanoparticles and Aβ_1–40_. We speculate that sequences containing β-sheet or α-helices in the hydrophilic domain may produce reversible structural change due to a pH change process. The β-sheet conformation has been assigned to the Aβ-oligomers, and a direct correlation between the β-sheet formation and Aβ concentration, which reaches a maximum around pH 5.4 for Aβ_1–40_, is known [41,42]. Hilblich *et al.* reported that a dimer is the predominant species at pH 7.0 and at physiological concentrations for Aβ [43].

### 2.3. The Protein Dependent Reversible Process

**Figure 6.**
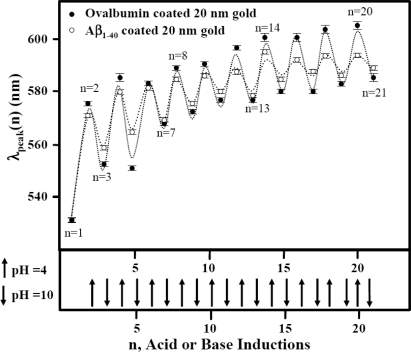
A demonstration of the color change reversibility seen in Aβ_1–40_ coated 20 nm gold colloidal particles and ovalbumin coated 20 nm gold colloid. The upward and downward arrows indicate an injection of HCl and NaOH, adjusting the pH of the solution to pH 4 and pH 10, respectively. The dashed lines indicate the values predicted by Equation 3.

We have compared the reversible process between Aβ_1–40_ and ovalbumin coated 20 nm gold colloid. In order to clarify the protein dependence for the reversible process, the observed reversible shift in λ_peak_ for Aβ_1–40_ or ovalbumin coated 20 nm gold colloid is shown in Figure 6.

The wave peak of each acid or base addition operation, λ_peak_(n), is represented by using Equation 3. (The parameters A, B, C, D, and E are shown in Table 1.) While the values of λ (1) were around 530 nm for both cases, the peak position started differ even at n = 3. Parameter B is a measure of the average peak wavelength at each induction, which converges at n = ∞. Therefore, a larger value of B means that the spectrum converges to a structure stabilized under acidic conditions by exhibiting more red shift at a relatively early induction number (a small value of n). The parameter C implies an induction number dependence of parameter B. Our result indicated that both cases show similar B and C parameters. The pre-exponential factor D represents the half value of the maximum difference of peak wavelength between pH 4 and pH 10 (*i.e.*, the difference between λ_peak_(pH = 4) and λ_peak_(pH = 10) is given by 2 × D nm). The D parameter for ovalbumin is larger than that of Aβ_1–40_, and this is consistent with the larger peak value shift in ovalbumin. Lastly, the parameter E exhibits how fast the oscillation character dies down (*i.e.*, a damping index). The parameter E has most significant difference between ovalbumin and Aβ_1–40_ among all parameters. A damping factor of amplitudes was larger in Aβ_1–40_ coated gold colloid by a factor of approximately 3.5. This indicates that the reproducibility of the structure over the colloid surface is higher for ovalbumin proteins. It can imply that the segments of ovalbumin which are responsible for the reversible structure are more resistant to the acid. This may also mean that the conformation of Aβ_1–40_ located on the gold colloid surface was more affected by the surface field (most likely electrostatic interaction) resulting in less freedom for Aβ_1–40_ to repeat the conformational change. From the fact that the entire peak is shifting toward a longer wavelength from the original starting value, protein may be unfolded (or denatured) by acid, and Aβ_1–40_ can be said to be denatured more easily by the acid.

While a specific structure of Aβ_1–40_ monomers assembled over 20 nm gold is not determined from this study, comparison of 20 nm gold colloid coated with Aβ_1–40_ and ovalbumin can hint some differences in self-assembled conformations between Aβ_1–40_ and ovalbumin. Our study revealed existence of a reversible structure observed only at an interfacial environment over gold nanocolloidal surfaces. Under no presence of gold colloidal particles, Aβ_1–40_ was reported to form the β-sheet conformation consisting of the Aβ-oligomers around pH 5.4 [41,42]. This is true for ovalbumin treated by base, and a predominant β-sheet conformation was observed. It was also determined that base treated ovalbumin exposed hydrophobic segments of the proteins [44].

### 2.4. The AFM Study

**Figure 7.**
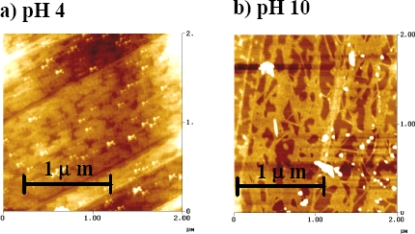
The AFM images of Aβ_1–40_ conjugated on the surface of 20 nm gold colloid on the surface of graphite. The condition of deposited solution was (a) pH 4 and (b) pH 10.

We prepared 20 nm gold colloidal particles and Aβ_1–40_ coated 20 nm gold colloidal particles at pH 4 and pH 10, corresponding to the samples at n = 2 and n = 3 in Figure 5, respectively. A film over either graphite or mica plate was created, and the image of this film surface was examined by Atomic Force Microscopy (AFM) under tapping mode. In Figure 7, the images collected for the graphite plate are shown. The graphite surface exhibited a very low sticking coefficient for gold colloid, resulting in segregation of the gold particles above the film surface. However, it clearly shows a different conformation of the layer beneath the gold colloid particles. The non-continuous layer seen at pH 4 may be attributed to a β-pleated sheet. The average size of the gold particles was founded to be approximately 40 nm in diameter. While we did not detect strong evidence of the gold colloid’s aggregation, the repulsive interaction of the graphite surface was considered to dominate at the surface so that the interaction between gold colloids causing aggregation was quenched. It was also observed that the Aβ_1–40_ was attached to the surface rather than conjugating to the gold colloidal surfaces. The conjugated Aβ_1–40_ was suspected to act like a mediator for the aggregation of gold colloids, but the clustering observed in the solution condition was not reproduced over the graphite surface. At pH 10, we observed a fiber like formation of the proteins with gold colloids located around the edge or surface of the network of the proteins. Under this condition, we found larger sizes of gold colloid particles (average of 55 nm) compared to those found in pH 4 (average of 40 nm). However, this is not consistent with the general consensus of gold colloids aggregating under the acidic condition. We suspect that the hydrophobic portion of the Aβ_1–40_ (i.e., sequences of 17 to 40) was used in attaching over the surface, and the hydrophilic tail (i.e., sequences of 1 to 16) was used for attaching the gold colloids. At the basic condition, the group possessing the positive charge (i.e., arginine, histidine, and lysine) must be quenched, and the part with the negative charge (i.e., aspartic acid and glutamine) may contribute to electrostatic interactions with the cluster of gold colloids.

**Figure 8.**
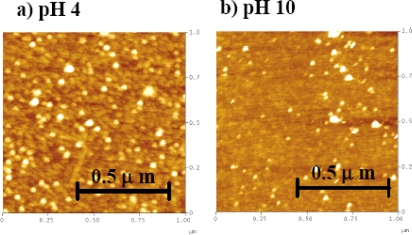
The AFM images of Aβ_1–40_ conjugated on the surface of 20 nm gold colloid on the surface of mica. The condition of deposited solution was (a) pH 4 and (b) pH 10.

We also collected the AFM images on a mica surface for the solutions prepared at pH 4 and pH 10 (See Figure 8). At pH 4, we were able to observe a film of granular morphology with limited fiber-like structures of the protein with gold dispersed along the network. However, this protein/gold granular morphology was not clearly observed for pH 10. For both pH 4 and pH 10 conditions, the average particle size of the gold colloids was 30 nm and we did not find strong evidence of aggregation of the gold colloids. The mica surface provided a high sticking coefficient for the solution, so the original molecule arrangement in the solution may remain over the mica surface better than over the graphite surface.

The AFM images show different morphologies of Aβ aggregates coated with gold colloids on mica and graphite. This may be due to different properties (hydrophobic /hydrophilic) of the surface environment. The AFM study provided general, but not a conclusive, morphology of the protein around the gold colloidal particle. However, the aggregation of the gold colloids was not clearly observed, therefore it did not support the cause of color change observed in the reversible process between pH 4 and 10.

### 2.5. The TEM Study

**Figure 9.**
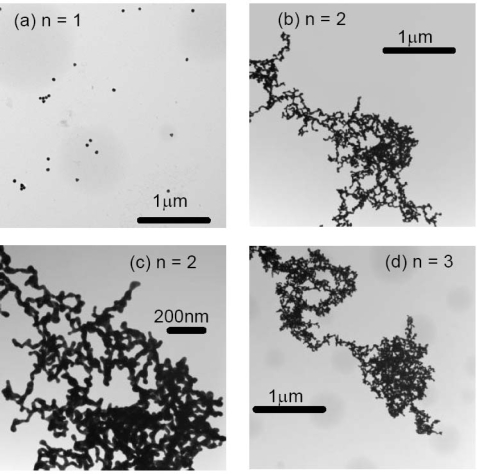
The TEM images of 20 nm gold colloids (a) n = 1 (pH 7), (b) n = 2 (pH 4), (c) n = 2 (pH 4), and (d) n = 3 (pH10).

**Figure 10.**
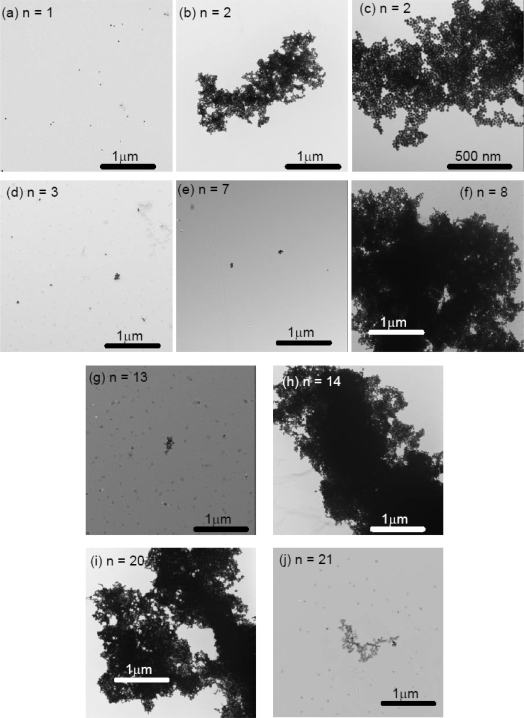
The TEM images of ovalbumin coated 20 nm gold colloids (a) n = 1 (pH 7), (b) n = 2 (pH 4), (c) n = 2 (pH 4), (d) n = 3 (pH 10), (e) n = 7 (pH 10), (f) n = 8 (pH 4), (g) n = 13 (pH 10), (h) n = 14 (pH 4), (i) n = 20 (pH 4), and (j) n = 21 (pH 10).

**Figure 11.**
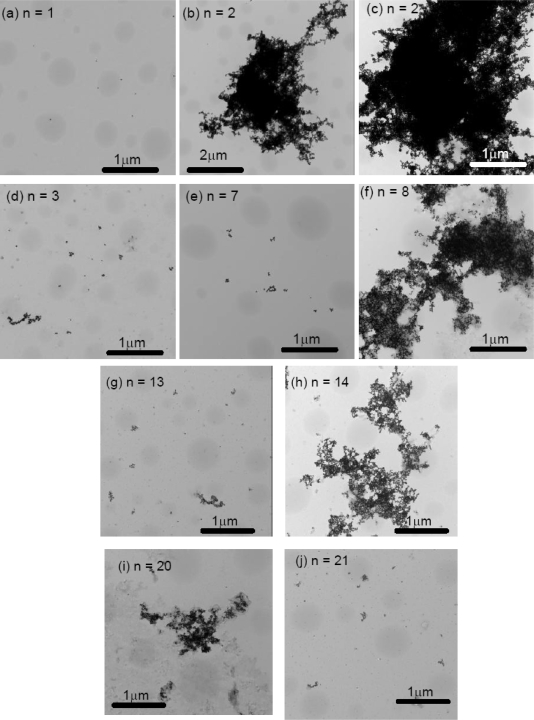
The TEM images of Aβ_1–40_ coated 20 nm gold colloids (a) n = 1 (pH 7), (b) n = 2 (pH 4), (c) n = 2 (pH 4), (d) n = 3 (pH 10), (e) n = 7 (pH 10), (f) n = 8 (pH 4), (g) n = 13 (pH 10), (h) n = 14 (pH 4), (i) n = 20 (pH 4), and (j) n = 21 (pH 10).

The Transmission Electron Microscopy (TEM) images were collected for 20 nm gold colloid alone, 20 nm gold colloid coated ovalbumin, and 20 nm gold colloid coated with Aβ_1–40_ that were cycled between pH 4 and pH 10. While gold colloid 20 nm solution did not exhibit any repeating features in color change, the 20 nm gold colloid ovalbumin and Aβ_1–40_ showed quasi-repetitive color change as pH was altered externally between pH 4 and pH 10. Thus, we collected TEM images for n = 1, 2, and 3 in the case of gold colloid alone (Figure 9) and for n = 1, 2, 3, 7, 8, 13, 14, 20, and 21 for the gold colloid coated protein solutions (Figures 10 and 11).

The TEM image analysis was performed by converting the image to data of pixel coordinate and corresponding color index. We set the threshold in color index to recognize the group of pixels corresponding to the gold particles and the average size of the gold particles, ratio of the area occupied by the gold particles (occupancy, %), and the number of the gold particles were calculated. These values are given in Table 2.

We observed a clear correlation between spectroscopic (or colorimetric) indication of the solution and the conformation of the gold colloids observed in TEM. Generally speaking the gold particles were dispersed for the initial (pH = 7) and basic conditions, and the gold particles aggregated at acidic condition. This phenomena was quantified by the occupancy rate of the area by the gold particles as tabulated in Table 2. For example, the occupancy rate of the Aβ_1–40_ coated 20 nm gold particles at n = 2 is ~ 73 % and that for n = 3 is only ~ 0.6 %. Even at the basic condition, we observed a small clusters of gold particles that consist of 10 or 20 gold particles as n increases. However, the cluster size never approached the acidic condition where clusters contained thousands of gold particles.

The aggregate of the 20 nm gold particles alone at pH 4 shows a sign of the destabilization of the colloid such that each spherical colloid particle could not be identified. On the other hand, the particles in the aggregates formed by the protein-coated 20 nm gold colloid maintained their original spherical shape. In some situations we could spot the spacing between each gold particle. This implies that the protein coat of the gold particles mediates aggregation. It also implies that the protein coated gold particles are stable even at the acidic condition, since they avoid direct surface contact with the acidic solution. For the Aβ_1–40_ coated 20 nm gold colloid, the number of particles that form aggregates decreased as the number of cycles increases. This may imply that the Aβ_1–40_ underwent irreversible structural changes as the cycle number was raised. Thus, the number of gold particles per aggregate dropped in the low pH 4.0 samples (compare for example Figure 11b and Figure 11i). This presumably also explains the λ_peak_ shifts toward larger wavelengths after several cycles of pH changes. The ovalbumin coated 20 nm gold particles formed aggregates that consisted of a relatively high number of gold particles. We may conclude that the degree of denaturization by the acid was less in ovalbumin than in Aβ_1–40_. This difference in denaturization was also consistent with what was observed in the quasi-reversible process of the color change. There, the amplitude of the reversibility (difference of the peak position between pH 4 and pH10) was larger in ovalbumin coated gold colloid than that of Aβ_1–40_ coated gold colloid. (See Figure 6).

After a large number of cycles, we observed smear like backgrounds which did not possess the beadlike shape of the gold particles. The substances observed in the background was presumably the salt formed during the repetitive addition of NaOH and HCl. As the n number increases the concentration of this background substance seems to increase and the aggregates seem to become less distinct. The existence of the salt may interrupt the conjugation of the protein over the colloidal surface. The observation of this background substance may indicate the inhibition of protein conjugation due to the salt formation as well as the major reason of the decrease in the amplitude of the reversibility.

## 3. Experimental Section

The gold colloidal nanoparticles with diameter of 19.7 ± 1.1 nm (7 × 10^11^ particles/mL) were purchased from Ted Pella Inc. (Redding, CA, USA). These the gold colloids were formed by a Frens derived citrate reduction method possessing traces of citrate < 0.00001%, tannic acid < 0.0000001% and potassium carbonate < 0.00000001%. Ultra-pure amyloid beta Aβ_1–40_ peptide (MW: 4329.9 Da) was obtained from American Peptide Corp. (Sunnyvale, CA, USA) and stored at −12 °C. (The reported purity was 95.0% by HPLC) The Grade II chicken egg albumin (ovalbumin, MW: 44.3 kDa) was purchased from Aldrich Co. (St. Louise, Missouri, USA) In the procedure, any water used was purified to more than 18 MΩ using a Milli-Q water system (Millipore). The stock solution of 100 μM all Aβ was prepared at approximately 18 °C. The amount of Aβ was determined by UV absorption at 280 nm (absorbance of Tyrosine at 275 nm, ε_275_ = 1,390 cm^−1^ M^−1^) [45]. The concentration of gold colloid particles in this experiment was 0.19 nM and the concentration of the protein solution was 0.19 μM.

After protein was vigorously mixed with the 20 nm gold nanoparticle solution, the samples were left for at least one hour and the pH was then adjusted between 4 and 10. The pH value of each sample was directly measured in the stirring cell using a micro pH electrode with an accuracy of ± 0.002. The pH change from 7 to 2 was by drop-wise addition of hydrochloric acid (HCl) and that of between 7 and 10 was by addition of sodium hydroxide (NaOH). Since the commercial gold nanoparticles contained some residual acid, resulting in a buffer against basic conditions, the solution pH was monitored for a long time, and additional base was continuously added as required for the range between pH 8 and pH 10.

The AFM tool is Digital Instrument Nanoscope III (Veeco, Plainview, NY, USA) and the tip is Budget Sensor BS-Tap300Al. As for the substrate, we used either graphite or mica plate. About one μL of sample solutions were dropped over the sample plate and the surface was purged with nitrogen gas after two minutes.

The TEM samples of the uncoated and ovalbumin coated colloids were made with carbon coated copper grids (Electron Microscopy Sciences, Hatfield, PA, USA). For the protein solution 8 μL of protein was mixed with 40 μL of gold colloid and one μL of solution was pipette onto the surface of the grid. After two minutes to allow the sample to bind to the grid, excess solution was removed from the grid with filter paper. However, because the Aβ_1–40_ coated gold beads did not bind evenly to the carbon-coated copper grid, we instead used Formvar -coated grids for these samples. Formvar was cast onto microscope slides, floated off onto water, and grids were applied to the film. Samples were examined with a Morgagni model 268 TEM (FEI Co., Hillsboro, OR, USA) operated at 80 kV. Images were taken at a nominal magnification of 28,000 or 71,000 on a model XR-40 four megapixel CCD digital camera (AMT).

## 4. Conclusions

A quasi-reversible color change between pH 4 and pH 10 took place with Aβ_1–40_ or ovalbumin coated 20 nm gold colloids. Spectroscopic features corresponding to the pH induced color change were investigated at a microscopic level using AFM and TEM The AFM image on graphite surface exhibited the sheet-like formation of the Aβ aggregate, indicating the formation of a β sheet in our experimental conditions. The exposure to water can be considered to create the major morphology of the protein or gold colloid itself, implying that the presence of the water or solvent plays a key role in the conformation of the Aβ-gold colloid aggregation. Based on the TEM study, Aβ-conjugated gold colloids disperses at pH 7 and pH 10, but the gold colloids aggregate at pH 4. While the conformation of the protein was not confirmed, the observed gold colloids morphology difference between pH 4 and pH 10 must explain the color change (or spectral shift). While the acidic condition destabilized the gold colloid and deformed the spherical colloidal shape, the protein conjugated gold colloids remained in individual spherical shape. This implies that the gold colloids are coated by the protein over their surfaces and the direct acid interaction was with the protein, causing denaturaization and possibly unfolding. It is clear that the pH induced conformational changes are sensitive to the interfacial environment. Our results provide a crucial implication of pH and corresponding conformation changes observed in fibrillogenesis that take place at the interfacial surface of human membranes.

## Figures and Tables

**Table 1.** The list of extracted parameters (A, B, C, D and E) with the use of Equation 3 for ovalbumin or Aβ_1–40_ coated 20 nm gold colloids.

	**A (nm)**	**A – D (nm)**	**B (nm)**	**C**	**D (nm)**	**E**
**Ovalbumin coated**	545(4)	531(4)	14(4)	0.42(7)	14(2)	0.02(1)
**Aβ_1–40_ coated**	542(2)	532(2)	21(2)	0.29(3)	10(1)	0.07(2)

**Table 2.** The list of occupancy of the gold particles in a given area and number of gold particles in each aggregate for 20 nm gold particles, 20 nm gold particles coated with ovalbumin and 20 nm gold colloid coated with Aβ_1–40_. The number of particles are shown only when the solution is acidic condition where the aggregates are formed.

	**Gold 20 nm**		**Gold 20 nm coated with ovalbumin**		**Gold 20 nm coated with Aβ_1–40_**	

n	Occupancy (%)	Number of gold particles per aggregate	Occupancy (%)	Number of gold particles per aggregate	Occupancy (%)	Number of gold particles per aggregate
1	0.37 ± 0.04	-	0.07 ± 0.03	-	0.06 ± 0.04	-
2	38 ± 4	350	55 ± 5	6,200	73 ± 1	24,000
3	27 ± 2	780	0.1 ± 0.04	-	0.6 ± 0.3	-
7			0.30 ± 0.02	-	0.6 ± 0.4	-
8			77 ± 3	19,000	31 ± 5	15,000
13			0.19 ± 0.01	-	0.4 ± 0.1	-
14			66 ± 5	22,000	13 ± 3	4,400
20			63 ± 4	25,000	7 ± 1	2,300
21			0.07 ± 0.03	-	0.5 ± 0.3	-
